# Clinical trials landscape in a lower-middle-income country (Pakistan)

**DOI:** 10.1017/cts.2023.701

**Published:** 2023-12-10

**Authors:** Hassan Mumtaz, Syed Muhammad Ali Haider, Fnu Neha, Muhammad Saqib, Abdullah Nadeem, Zoha Seikha

**Affiliations:** 1 Clinical Research Associate, Maroof International Hospital, Islamabad, Pakistan; 2 Public Health Scholar, Health Services Academy, Islamabad, Pakistan; 3 BMY Health, Lahore, Pakistan; 4 Ghulam Muhammad Mahar Medical College, Sukkur, Pakistan; 5 Khyber Medical College, Peshawar, Pakistan; 6 Dow University of Health Sciences, Karachi, Pakistan

**Keywords:** Clinical trial, Pakistan, overviews, developing country

## Abstract

In recent times, there have been calls from within the developing nations for increased ownership by governmental research bodies and universities of the priority research setting and research that aligns with national health strategies. This is a review paper of the studies that have been published on clinical trials in developing countries, with a focus mainly on Pakistan. The literature review used online databases such as PubMed, Scopus, and Google Scholar, World Health Organization (WHO) International Clinical Trials Registry Platform (ICTRP), and ClinicalTrials.gov trial registries to search for clinical trials conducted in Pakistan between January 2000 and December 2022 and analyzed. The results revealed that clinical research in Pakistan is hindered by a number of barriers, including a lack of funding, skilled personnel, and regulatory issues. Lack of funding is a common obstacle, and the majority of funding for clinical trials originates from Western countries or pharmaceutical companies established in the West. In conclusion, clinical studies in developing countries, especially in Pakistan, are hindered by a plethora of barriers, and to improve the current state, increasing funding, streamlining ethical approval procedures, simplifying regulatory systems, addressing cultural and religious concerns, and participating in global efforts to bridge the gap in health-based research are crucial.

## Introduction

Clinical trials play a pivotal role in ascertaining the effectiveness, safety, and adverse effects of novel therapeutic modalities before their widespread implementation within the general populace. The compounded burden of disease prevalent in developing nations underscores the critical imperative for conducting clinical research aimed at identifying and implementing cost-effective and innovative treatment regimens. Such research endeavors serve as guiding principles for healthcare practitioners operating within resource-constrained environments, characterized by limited medical and surgical capabilities. Alarming disparages in the distribution of clinical trial sites were revealed in a comprehensive study conducted in 2018, wherein an overwhelming 83% of trials were conducted exclusively within 25 high-income nations, while a mere fraction, less than 5%, encompassed 91 lower-middle or low-income countries (LMICs). Despite the disproportionately high disease prevalence observed within LMICs, an unfortunate dearth of substantial funding and research initiatives exists, exacerbating the persistent gap in healthcare advancements among these nations [1]. Practical and pragmatic clinical trials (PCTs) play a role in healthcare decision-making. These trials are deliberately designed to provide answers to questions. They stand out for their focus on interventions that are clinically relevant inclusion of participants from practice settings and comprehensive data collection across a wide range of health outcomes. The limited availability of PCTs is primarily due to funding constraints from sponsors of research. PCTs have a role in generating useful insights for healthcare choices making them essential, for addressing real-world healthcare inquiries [2]. For example, a clinical trial conducted in northern India suggests that the Diabetic Yoga Protocol intervention might improve the metabolic state of high-risk people in terms of glucose tolerance and cholesterol levels, which could be explained in part by a reduction in abdominal obesity. The study emphasizes the potential impact of yoga intervention in improving the cardiovascular profile in a high diabetes-risk population in real time [3].

Developing nations (a nation with an average income less than industrial nations) constitute a significant proportion of the global population. These countries are home to a vast majority of the worldwide burden of disease, the majority of which are preventable infectious diseases [4]. The healthcare systems in developing nations are in dire need of evidence-based guidance to make informed decisions about the most efficient and cost-effective interventions. The paucity of resources in these countries paradoxically amplifies the requirement for reliable healthcare evidence to aid in the prioritization of their limited resources [5]. On the other hand, diseases that are of relevance to high-income nations are investigated in clinical trials at a rate that is seven to eight times higher than the diseases whose burden is primarily borne by developing nations [6].

## Methods

This is a review paper with an original search as well as a review based on a literature review of studies and articles that have been published on clinical trials in developing countries, with a focus mainly on Pakistan. The literature review used online databases such as PubMed, Scopus, and Google Scholar. Moreover, original research from the World Health Organization (WHO) International Clinical Trials Registry Platform (ICTRP) and ClinicalTrials.gov trial registries were searched for clinical trials conducted in Pakistan between January 2000 and December 2022. The data from other registries were searched with the same procedures that were used to search the leading registries. The data were analyzed with the results discussed in this paper. The data collected included the type of trial, funding source, therapeutic area, phase of the trial, and province where the trial was conducted. The results were analyzed and presented in frequency and pie charts. The data were analyzed to identify patterns and trends in the clinical trial landscape in Pakistan, specifically regarding the phase of the trial, study type, and location of the trials. In addition to the ICTRP search, we also conducted a manual search of relevant journals and conference proceedings to identify any additional clinical trials that may have not been included in the WHO registry. The search was not limited by year or language. The data were analyzed using descriptive statistics and presented in frequency and pie charts to provide an understanding of the current landscape of clinical trials in Pakistan.

## Results

**Figure 1.**
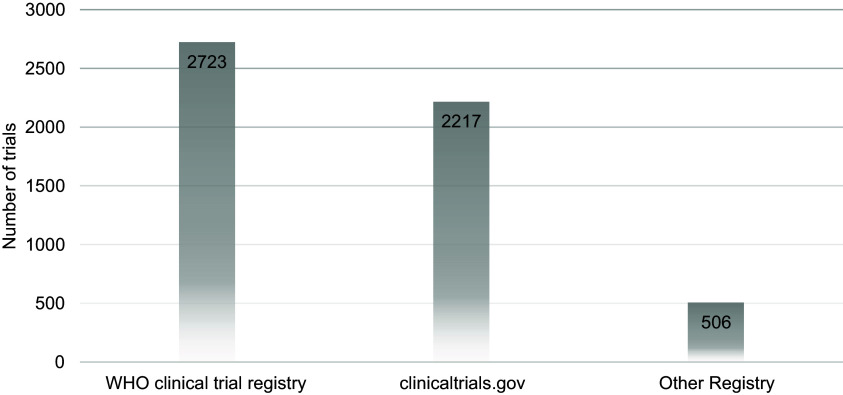
Registration sources of clinical trials conducted in Pakistan between January 2000 and December 2022; number of trials in each source are described on the corresponding bars.

**Figure 2.**
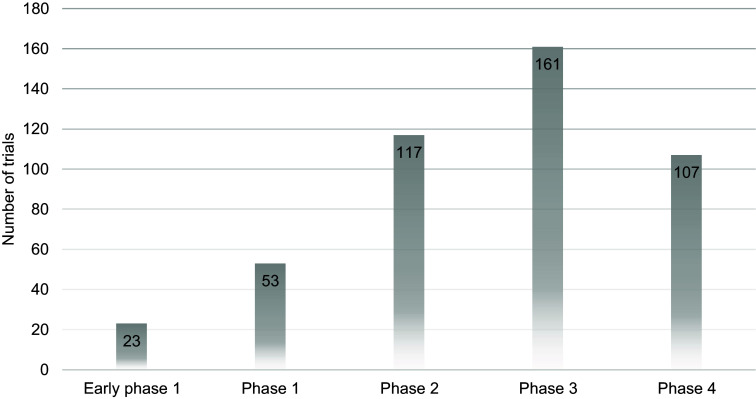
Phase-wise distribution of clinical trials in Pakistan; the number of trials in each phase is described on the corresponding bars.

**Figure 3.**
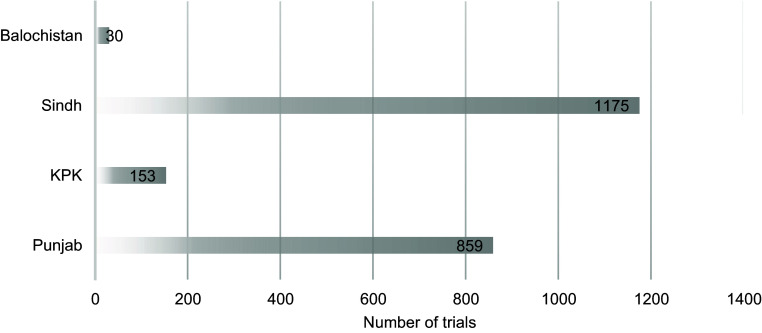
Provincial distribution of clinical trials in Pakistan; the number of trials conducted in each province of Pakistan is described on the corresponding bars; KPK = Khyber Pakhtunkhwa.

**Figure 4.**
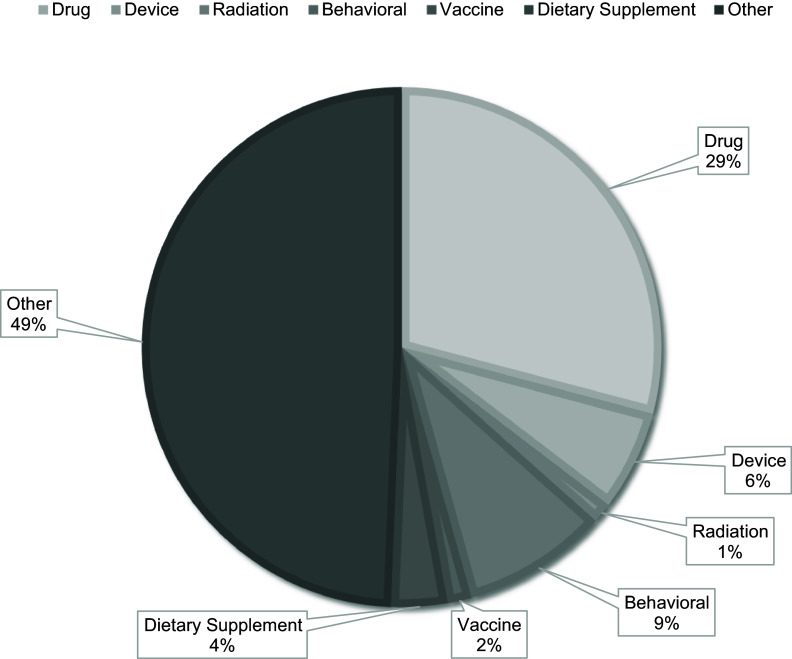
Pie chart showing different types of trials conducted in Pakistan expressed as a percentage; any trial that doesn’t fit into or didn’t specify type is depicted under the “other” group.

The WHO clinical trial registry contained information on 2723 studies from January 2000 to December 2022. The data from 2217 of the 2723 studies were also present on ClinicalTrials.gov, while the remaining were from other registries. Data regarding the phase of the trial were not reported for more than 79% of the trials in Pakistan. The majority of the trials were phase III trials (in these studies, new therapies are compared to the best currently available treatment (the standard treatment)) based on drugs, and they were reported to be of interventional type. Pharmaceutical industries only made up of about 9% of the sponsors, while the majority of the rest of trials were sponsored by universities. Details regarding clinical trials can be found in Table 1, their registration sources in Figure 1, the phase-wise distribution in Figure 2, the provincial distribution of clinical trials in Figure 3, and the trial type distribution in Figure 4.

**Table 1.** Summary of clinical trial details in Pakistan; NIH = National Institutes of Health, Pakistan

Phase of study	Number
Early Phase 1	23
Phase 1	53
Phase 2	117
Phase 3	161
Phase 4	107
Phase not applicable	1756
**Study type**	
Interventional	1734
Observational	483
**Provincial location**	
Punjab	859
Sindh	1175
Baluchistan	30
Khyber Pakhtunkhwa	153
**Sponsors**	
NIH and other US funding agencies	33
Pakistani universities	1829
Pharmaceutical industries	200
**Intervention type**	
Drug	635
Device	136
Radiation	27
Behavioral	197
Dietary supplement	76
Vaccine	33
Other	1075

## Discussion

In recent times, calls for increased ownership of priority setting and research that aligns with national health strategies in developing nations have been made. This is seen as a crucial step toward addressing the healthcare needs of these nations more effectively and sustainably [7,8]. Most developing countries lack the resources and trained staff which puts them at a loss when it comes to clinical trials. However, with new reforms in public health, a change has been observed in countries like Pakistan. The pandemic brought on new challenges which were tackled by an era of research like no other. The number of trials increased after the pandemic with the focus being on the impact of COVID-19 on the healthcare population and students. In-house trials at tertiary care centers include recent trials comparing different operative approaches to standard procedures such as cholecystectomy. The scope for developing trials is very encouraging with more innovative studies being developed. A study conducted in 2018 highlighted a significant disparity in the distribution of clinical trial sites, with 83% of trials conducted in 25 high-income countries and less than 5% in 91 lower-middle or low-income countries (LMICs) [1]. This highlights a significant imbalance in the distribution of resources and opportunities for clinical trials in different parts of the world, with a disproportionate focus on high-income nations at the expense of LMICs [9]. Clinical research is hindered by a plethora of barriers, including a lack of financial resources, a lack of skilled personnel, and regulatory and administrative issues. Alemayehu *et al*. [10] reported that lack of funding is a common obstacle and highlighted that the majority of funding for clinical trials originates from Western countries or pharmaceutical companies established in the West. Clinical research in developing countries is progressing at a very steady rate. Over time multiple efforts have been made globally to reduce the inequity in health-based research to transform health with limited resources, one of which includes the European and developing countries’ clinical trial partnership which is a project aimed at bridging this gap [11]. The only limitation that researchers come across is the limited infrastructure and logistical issues. This has been observed in other developing countries such as Vietnam, and constant quality enhancement is required in this field [12]. The lack of funding for clinical trials in developing countries such as Pakistan may stem from the fact that there is a limited budgetary allocation for this purpose. The lack of emphasis on clinical trial research in medical schools and teaching hospitals, and the absence of research-based higher educational institutions, have led to a shortage of skilled personnel. Furthermore, individuals with specialized training or experience in clinical trials often opt to work abroad due to greater opportunities, resulting in brain drain in their countries [1,10]. Unnecessary delays in ethical approval procedures and complex and unreasonably strict government regulatory systems have further impeded progress [13]. Additionally, certain cultural and religious beliefs that create fear of exploitation among the general population have also hampered advancement [14].

**Figure 5.**
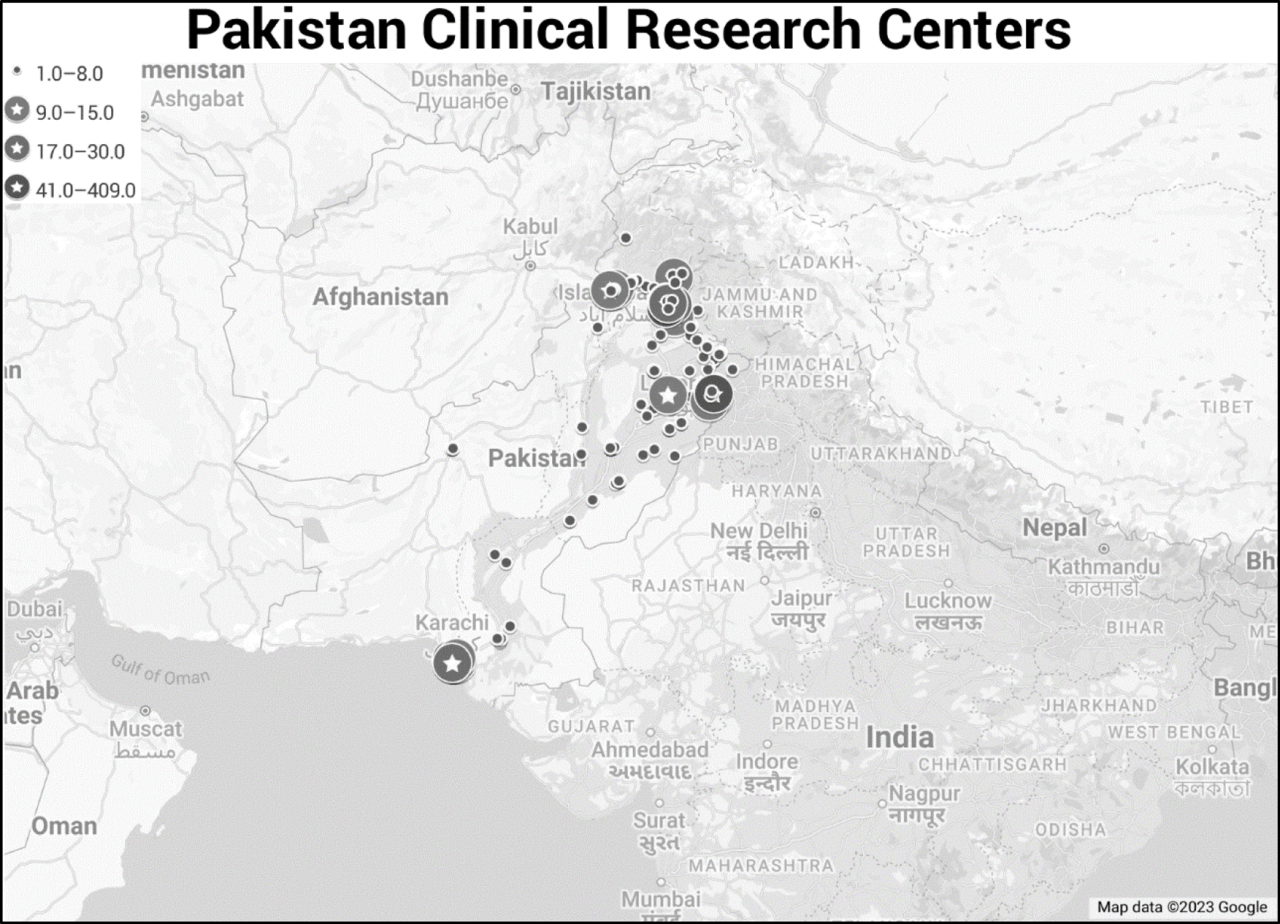
Geographical distribution of clinical trial centers in Pakistan as registered in US ClinicalTrials.gov; this map has been adopted with written permission from the map owner (Anwar B); see [18] for further details.

The scholarly community in Pakistan has been actively engaged in the conduct of clinical trials within the country. With the initiation of the first trial dating back to 1992, a total of 508 clinical trials have been recorded on ClinicalTrials.gov, a registry that was established in 2000 and has since become the premier repository for clinical trial information globally [15,16]. Since then, upward of 2700 registered clinical trials have been performed in Pakistan according to our results. Pakistan is a lower-middle-income nation and faces the challenge of a significant proportion of its population living below the poverty line, with 24.3% of the population being affected. The healthcare sector in Pakistan receives limited allocation in the national budget, accounting for less than 3% of the total. According to the WHO, the distribution of physicians, nurses, and midwives per 10,000 populations in Pakistan is 8.1 and 10.6, respectively, which is below the regional average [17]. Despite the constraints of limited resources and an inadequate framework, academic institutions throughout the country have been actively engaged in the promotion and production of clinical research. Since the early 1990s, clinical trials have been steadily increasing in number. Several hospitals and universities have state-of-the-art clinical trial units, offering various educational programs and courses to enhance the quality of research produced in Pakistan [14]. In line with our results, the province of Sindh is home to the largest number of clinical trials conducted in Pakistan. This has also been confirmed by Anwar *et al*. [18], and they depicted clinical research centers in Pakistan as shown in Figure 5. This represents the number of clinical research projects, both observational and interventional in Pakistan, that have been registered on ClinicalTrials.gov. The data collection sites have been identified and categorically divided according to the number of projects conducted at each location. The red circles indicate sites that have conducted between 1 and 8 projects, while the yellow circles denote sites that have conducted between 9 and 16 projects. The orange circles signify sites that have conducted between 17 and 30 projects, and the green circles represent sites that have conducted more than 30 projects.

This visualization serves as a useful tool for understanding the distribution and concentration of clinical research endeavors across various sites. COVID-19 has had an impact on clinical research worldwide as well as in Pakistan. For instance, Park *et al*. [19] in a recent study reported that the landscape of COVID-19 clinical trials has been marred by a lack of geographical diversity, with the majority of ongoing trials concentrated in high-income countries such as the European Union, the United Kingdom, and North America. This is particularly concerning as LMICs, which are often disproportionately affected by pandemics due to poverty and fragile healthcare systems, are underrepresented in these trials. To address this imbalance, it is crucial to expand the evaluation of cost-effective and scalable COVID-19 interventions in resource-poor settings. The power imbalance between researchers from high-income countries and those within LMICs, as well as the short-term nature of many clinical trials, have contributed to this issue. To rectify this, researchers from high-income countries should engage in true collaborative partnerships with LMIC researchers, and funding decisions should focus on creating long-term support for LMICs. Platform trials, which allow for the examination of multiple questions over time in a single large trial, can provide robust and conclusive evidence and help to build sustainable capacity and infrastructure in LMICs. Furthermore, capacity development should not be limited to frameworks and systems alone but should also encompass the creation of funding models that can recruit and retain dedicated expert reviewers. Only through such efforts, the gap in representation can be closed and LMICs can be meaningfully included in the global fight against COVID-19 [19].

According to a recent analysis by Siddiqui *et al*. [17] which is in line with our observations, interventional clinical trials have been conducted more frequently than observational trials in Pakistan, with 77.2% of trials being interventional and 22.8% being observational. The majority of these trials have been conducted in recent years, specifically between 2010 and 2019, representing 65.5% of interventional trials and 71.5% of observational trials with the most common type of clinical trial reported to be drug interventions, comprising 41.4% of all trials. According to their analysis, more than half of all trials were conducted in the last decade [17]. Our studies also indicate that pharmaceutical industries made up a small portion of funding for clinical trials and that most of the trials conducted were phase III trials. It is estimated that clinical trials can be more than a 150 million US dollar industry in the next 5 years and this can be a wonderful opportunity for the industry to take part in the scientific process and increase the pharmaceutical industry’s share in doing clinical trials in Pakistan [20].

Despite the challenges, LMICs which include Pakistan showed the highest annual growth rate in clinical trials in the period 2006–2012 (14.7%) compared to the USA, high-income countries, upper-middle-income countries, and low-income countries [1,9]. The presence of clinical trials offers a range of therapies for patients and also assists physicians in targeting new biochemical components for various pathologies. According to WHO, Pakistan is among the top five countries with the highest prevalence of TB, hepatitis B and C, and HIV [21]. Despite these being highly infectious diseases, the spread has been curbed globally with the use of updated drug regimens. To combat this spread, it is pertinent to develop trials specific to this particular ethnicity to better tackle such concerns. In addition to infectious illnesses, highly preventable chronic illnesses are also highly prevalent in Pakistan.

### Challenges

Researchers in Pakistan face a plethora of challenges, particularly insufficient funding and resources. This has resulted in a lag in research output when compared to other developing nations in the region. Despite these obstacles, various efforts have been made to address these issues. In 2002, the International Conference on Harmonization-Good Clinical Practice (ICH-GCP) guidelines were adopted as the gold standard for conducting clinical trials. Additionally, a committee was established to oversee the implementation of Good Clinical Practice (GCP). The establishment of the Drug Regulatory Authority Pakistan (DRAP) provided a framework for the approval and regulation of individual clinical trials. However, a persistent problem remains in the form of inadequate funding for research studies [14,17]. Furthermore, there is currently no local standard trial registry in Pakistan, requiring trials to be registered with ClinicalTrials.gov. This has been highlighted by multiple researchers and has been acknowledged as an issue in need of resolution [22,23]. Another aspect to consider is the ethical standard of clinical trials in Pakistan. The country lags in terms of literacy, with a current estimated rate of 58%, and even lower for females at 48%. This raises concerns about informed consent and its role in clinical trials, especially in the context of a predominantly patriarchal society. Additionally, the absence of a regulatory body to supervise and monitor clinical trials is a cause for concern. Studies have also been conducted to assess the attitude of junior doctors and physicians-in-training regarding clinical research. These surveys have shown that trainees with prior education and experience in research are more likely to be involved in conducting studies. Limited time, poor research infrastructure, and inadequate funding opportunities were identified as major hurdles. Although funds are provided by organizations such as the Higher Education Commission (HEC), Pakistan Medical and Research Council, and Pakistan Science Foundation, these resources mainly fund basic research studies and are insufficient for large-scale clinical trials [14].

### Future Recommendations and the Way Forward

To improve the current state of clinical trials in Pakistan, several directions can be taken in the future. First, increasing funding for clinical trial research in Pakistan is crucial. This can be achieved by allocating more budget to this purpose by the government, as well as encouraging funding from private and international sources. Additionally, emphasis on clinical trial research in medical schools and teaching hospitals need to be increased, as well as the establishment of research-based higher educational institutions. This will help to address the shortage of skilled personnel in the country. To address the logistical and infrastructural challenges, the government can also work on streamlining ethical approval procedures and simplifying government regulatory systems to make them more efficient. Furthermore, efforts can be made to address cultural and religious concerns that may allay the fear of exploitation among the general population. Lastly, Pakistan can also benefit from global efforts to bridge the gap in health-based research in developing countries. One example of such an effort is the European and developing countries' clinical trial partnership, which aims to support the conduct of high-quality clinical trials in LMICs [11]. By participating in such initiatives, Pakistan can access the necessary resources, expertise, and opportunities that will help in conducting high-quality clinical trials in the country [24].

## Data Availability

The data used in this review were obtained from publicly available sources.

## Data sharing statement

The data used in this review were obtained from publicly available sources.
