# Clinical trials landscape in lower middle-income country (Pakistan) – CORRIGENDUM

**DOI:** 10.1017/cts.2024.22

**Published:** 2024-02-22

**Authors:** Hassan Mumtaz, Syed Muhammad Ali Haider, Fnu Neha, Muhammad Saqib, Abdullah Nadeem, Ammad Fahim, Zoha Allahuddin

The above review published with mistakes in some author names and affiliations. One author name was not included in the author list at all.

Hassan Mumtaz^1,2^, Syed Muhammad Ali Haider^3^, Fnu Neha^4^, Muhammad Saqib^5^, Abdullah Nadeem^6^, Ammad Fahim^7^ and Zoha Allahuddin^6^

^1^Clinical Research Associate, Maroof International Hospital, Islamabad, Pakistan; ^2^Public Health Scholar, Health Services Academy, Islamabad, Pakistan; ^3^BMY Health, Lahore, Pakistan; ^4^Ghulam Muhammad Mahar Medical College, Sukkur, Pakistan; ^5^Khyber Medical College, Peshawar, Pakistan and ^6^Dow University of Health Sciences, Karachi, Pakistan ^7^Director Research, Maroof International Hospital Islamabad, Pakistan

The original review has been corrected online to rectify these errors.

See below for the authorship contribution statement, not included in the original review.

H.M.: Conceptualization, methodology, investigation, data curation, writing - original draft preparation, visualization, and supervision.

S.M.A.H.: Writing - original draft preparation, data curation, and methodology.

F.N.: Writing - review and editing, data curation, visualization, and methodology.

M.S.: Investigation, data analysis, writing - original draft preparation, visualization.

A.N.: Writing - review and editing, proofreading, methodology, and investigation.

A.F.: Conceptualization, methodology, data curation, writing - review and editing.

Z.A.: Writing - review and editing, data curation, and investigation.
